# Deliberation, context, emotion and trust – understanding the dynamics of adults’ COVID-19 vaccination decisions in Germany

**DOI:** 10.1186/s12889-022-14587-7

**Published:** 2023-01-19

**Authors:** Selina Dasch, Jonas Wachinger, Till Bärnighausen, Simiao Chen, Shannon A. McMahon

**Affiliations:** 1 Heidelberg Institute of Global Health, Heidelberg University Hospital, Heidelberg, Germany; 2 Department of Global Health and Population, Harvard T.H. Chan School of Public Health, Boston, USA; 3 Chinese Academy of Medical Sciences & Peking Union Medical College, Beijing, China; 4 Department of International Health, Johns Hopkins Bloomberg School of Public Health, Baltimore, USA

**Keywords:** Germany, Vaccines, COVID-19, Trust, Decision-Making, Emotions

## Abstract

**Background:**

Willingness to vaccinate against coronavirus disease 2019 (COVID-19), which is vital to successful vaccination campaigns, is wavering and suboptimal. In Germany, quantitative research highlighted concerns regarding the safety and efficacy of COVID-19 vaccines as barriers to uptake, but qualitative insights regarding individuals’ decisions about COVID-19 vaccines and how personal perceptions reflect or refute existing behavioral theories are lacking.

**Methods:**

To identify how individuals make COVID-19 vaccination decisions within real-life contexts, we conducted 33 semi-structured, in-depth qualitative interviews with individuals in Germany between March and April 2021 using maximum variation sampling, focusing on perceptions of COVID-19 vaccines. Analysis, informed by a framework approach, began in the field via debriefings and was amplified upon the conclusion of data collection.

**Results:**

Four interconnected themes (deliberation, context, emotion, trust) shaped respondents’ decisions about vaccination. Personal deliberation regarding benefits and risks of vaccines and perceptions of the broader social and political context sparked a spectrum of emotions that underpinned vaccination decisions. Trust in science and researchers emerged as a powerful protective factor facilitating the decision to get vaccinated even amidst a rapidly changing context and disconcerting information.

**Conclusions:**

Our findings add to ongoing debates about the breadth of vaccination decisions by highlighting how respondents are influenced by their perceptions of the political context and the emotional heft of their decisions. The role of cognitive evaluation, context, and emotions mirrors other decision-making frameworks, particularly the Risk as Feelings Theory. We extend on the elements of this theory by highlighting trust as a protective factor when making decisions particularly in highly uncertain contexts. Success of vaccination campaigns, more important than ever as new variants of COVID-19 emerge, is interwoven with an ability to bolster trust in science. Communicating public-health decisions and information about vaccines transparently without instilling fear offers promising chances to strengthen public trust in COVID-19 vaccines.

**Trial registration:**

German Clinical Trials Register (DRKS00024505).

**Supplementary Information:**

The online version contains supplementary material available at 10.1186/s12889-022-14587-7.

## Background

COVID-19 vaccines are considered a safe means to reduce COVID-19 incidence [1, 2] and to prevent severe morbidity or mortality [3]. However, reducing the burden of disease via comprehensive vaccination requires widespread willingness to be vaccinated [4], and such willingness has been wavering or suboptimal in several countries, including Germany [5, 6].

In this article we follow a definition of vaccine hesitancy as a “*delay in acceptance or refusal of vaccination despite availability of vaccination services*” [7]. This understanding acknowledges a continuum of vaccination attitudes, ranging from total vaccine acceptance to absolute vaccine refusal, with vaccine hesitancy being “*complex and context specific, varying across time, place and vaccines*” [7]. Several models have conceptualized vaccine hesitancy, often emphasizing contextual and psychological factors. The widely used 5C model, for example, outlines factors including complacency, constraints, confidence, collective responsibility (including social aspects) and calculation (information seeking) [8]. A review on influenza vaccine hesitancy emphasized slightly different factors that underpin vaccination intentions including sociodemographic, physical, contextual and psychological determinants [9].

In the context of COVID-19 vaccines, research has begun identifying reasons for or against COVID-19 vaccination uptake [10–12]. Personal protection [11], regaining a social life [10] and protecting others [10] serve as facilitators to vaccination whereas side effects [11], perceived low efficacy of the vaccine [11] and disconcerting (mis) information [13] underpin vaccine rejection. Uptake across vaccines often varies across countries and contextual circumstances [7, 12, 14]. For example, studies have shown that religious conviction sparked vaccine hesitancy in some contexts [15], yet potentially could facilitate vaccine uptake in others [16].

When asked about COVID-19 vaccines, survey respondents in Germany have highlighted concerns that vaccine-associated risks outweigh protective benefits [17]. Some subgroups including healthcare professionals who have managed severe COVID-19 cases [18], and older people who sense high personal susceptibility to illness [19], have, however, reported distinct pro-vaccination attitudes. In an overarching sense, studies in Germany and elsewhere have underscored the importance of trust in vaccines as a driver for vaccination uptake [6, 10], supporting claims that trust-building efforts are vital to successful immunization campaigns [20].

Extensive quantitative efforts undertaken globally have aimed to determine the breadth of COVID-19 vaccine hesitancy in several settings [21]. With notable exceptions [13, 22, 23] and one qualitative study from the German context [24], there is relatively less qualitative scholarship giving voice to individual perceptions of vaccines and intentions for vaccination uptake. Qualitative research captures person-centered views and lived experiences, which in turn can allow scholars and practitioners to identify insightful considerations in dynamic and evolving contexts [25]. Qualitative insights have informed the development of successful vaccine promotion materials, contributing to increased vaccination rates [26]. Thus qualitative research provides essential methods for understanding and responding to the ongoing COVID-19 pandemic. Leading voices in vaccination research have called for expanding qualitative investigations into public COVID-19 attitudes to inform vaccine confidence efforts [27].

This study addresses gaps in the literature by exploring COVID-19 vaccination decisions of a highly varied sample of the German population through in-depth qualitive insights of real-life experience.

## Materials & methods

### Study setting and political background

**Fig. 1:**
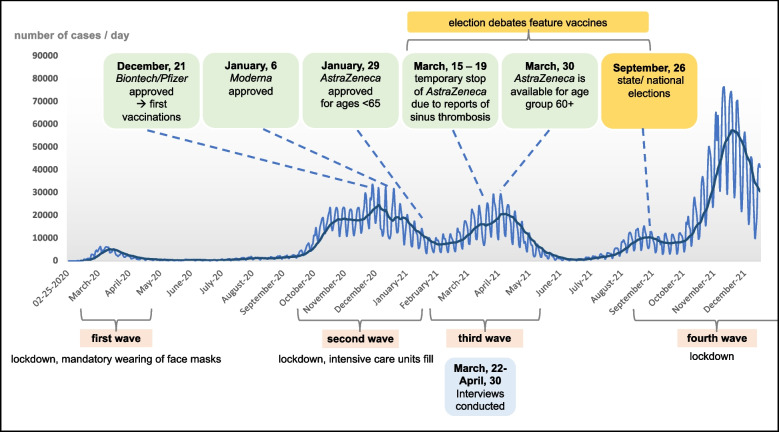
The broader study context amid COVID-19 (incidence, vaccine availability, political landscape) in Germany. Note: Own graphic with case numbers obtained from Robert Koch Institute [74]. Light blue line: COVID-19 cases in Germany per day. Dark blue line: Average of case numbers

On January 27, 2020, the first COVID-19 case was registered in Germany [28]. Due to rising incidence in March 2020, several infection control measures were adopted including: mandatory masks, school and store closures, and contact restrictions [28]. A first lockdown lasted from March to May 2020 (see Fig. 1 for a timeline and an overview of the COVID-19 pandemic and the broader context of data collection, including the vaccination campaign in Germany) [28]. Due to Germany’s political organization as a federation, general decisions were made at the federal level (parliament consisting of: “Bundestag” and “Bundesrat”), but implementation was decided at the state level by governing bodies within each of the 16 federated states (“Bundesländer”), resulting in regional heterogeneity [28]. Due to decreasing infection numbers, most restrictions were lifted during the summer of 2020 but reimplemented as incidence rose, sparking a second lockdown that lasted from November 2020 to April 2021 [28].

Despite overall high levels of approval toward infection control measures [29], protest movements occurred in Germany (often involving a movement referred to as “*Querdenken*”, a heterogeneous group protesting against contact restrictions, mask wearing, and COVID-19 vaccination) [30].

At the time of data collection (March and April 2021), Germany was in its second lockdown in the COVID-19 pandemic and COVID-19 incidence was increasing, marking the beginning of Germany’s third wave of infections [28]. Three vaccines against COVID-19 had been approved (Biontech/Pfizer on December 21, 2020; Moderna on January 6, 2021; AstraZeneca on January 29, 2021) but were available only to select priority groups (mainly those over age 75, individuals with certain diseases, and those working in selected fields) [31]. In total, about 15–20% of the German population had been vaccinated for the first time [32]. The rollout of AstraZeneca, which was initially only available for people younger than 65 years, was temporarily halted in Germany and other European countries due to emerging cases of sinus thrombosis after vaccine uptake, mainly among younger women [28, 33]. After a stop from March 15–19, the vaccine was reapproved in Germany but recommended mainly for people older than 60 years [28, 31, 33]. In terms of broader contextual realities, campaigns for German state elections were underway in two states, with national debates and their media coverage often centering on vaccines; national elections were expected to be held in September 2021 (where a new head of government would be chosen) [28].

### Design and sampling

For this in-depth qualitative study, we recruited respondents via Prolific Academic Ltd. (www.prolific.co), a platform allowing individuals interested in participating in scientific studies to register and scientists to contact them depending on their selection criteria and interests. Inclusion criteria included: fluency in German (to facilitate rich dialogue between interviewer and respondents), permanent residence in Germany (to capture a variety of experiences within one primary context), and a minimum age of 18 years as this is a legal regulation of Prolific Ltd. Drawing upon a maximum variation sampling approach [34], we prescreened respondents, aiming at a study sample with a variety of gender (male/female), age (< 50 years/> 50 years) and living conditions (rural/urban), as these characteristics are significantly associated with COVID-19 severity and mortality [35, 36].

### Data collection and analysis

A total of 36 individuals signaled interest to participate, with three respondents ultimately deciding against participation, citing scheduling problems (*n* = 2) or loss of interest (*n* = 1). The lead author, a medical student with a background in psychology and trained in qualitative interviewing, conducted all interviews via one-on-one video conferences using “Zoom” (*n* = 32) or “Skype” (n = 1) at a time of the respondents’ choosing. Interviews were conducted in German (n = 32) or English (n = 1, respondent was fluent in German but felt more comfortable in his mother-tongue), lasted 17–75 minutes and were based on a semi-structured interview guide (see Additional file 1) that focused on perceptions of COVID-19 vaccines, vaccine preferences and potential or actual barriers to vaccine uptake. Analysis started in the field through weekly debriefings [37], during which the team discussed organizational issues, reflexivity, and interview content including repeating patterns and opportunities to improve data collection. Saturation of themes was reached after 33 interviews and data collection concluded.

All interviews were audio recorded and transcribed verbatim by bilingual members of the research team. Drawing on a framework approach [38], the interviewer took copious notes during interviews, transferred them to a self-reflection sheet after each interview and combined them with an in-depth relistening process during which rapid transcription was used to extract text from especially salient items regarding vaccination decision-making. The lead author re-listened to all interviews to further bolster non-verbal cues such as pauses or emotional reactions and to heighten familiarity with the data. In the process of analysis and development of a thematic framework, we revisited the literature to define how salient themes in this study – namely, emotion and trust – reflected existing theory. We then developed a codebook by labelling identified themes and organizing them as codes and subcodes. The lead author applied the codebook to the entire dataset using Dedoose software [39] with oversight of the second and senior author; a process during which upcoming themes were adjusted and the framework was iteratively refined.

The study received ethical approval from the ethical review board of the Medical Faculty, Heidelberg University, Germany (S-041/2021). Respondents provided informed consent via an online consent-form as well as verbal confirmation at interview outset after receiving information about objectives of the study and the opportunity to ask questions. The amount of compensation was 10€, regardless of interview duration, and respondents received it directly after the interview via the Prolific platform.

## Results

A total of 33 respondents completed the interview, including males and females across a range of ages and living conditions (see Table 1).

**Table 1:** Respondent characteristics (*N* = 33)

Gender	Female *n* = 19
	Male *n* = 14
Age	Median = 35 (mean = 40)
	Range = 20–61
Living conditions	Urban *n* = 15
	Rural *n* = 18
Nationality	Bangladeshi *n* = 1
	British *n* = 1
	Dutch *n* = 1
	German *n* = 27
	Russian *n* = 1
	Turkish *n* = 1
	Zimbabwean *n* = 1
Educational background	Secondary education (“*Mittlere Reife”*, “(*Fach)Abitur”*) *n* = 16
	Graduate education / Bachelor’s degree *n* = 6
	Post-graduate education *n* = 11

Across all interviews, four major themes emerged regarding COVID-19 vaccination decisions: 1) Deliberation for and against vaccine uptake (benefits versus risks); 2) Social and political context; 3) Emotions towards the COVID-19 pandemic and the associated political and social processes; and 4) Trust in vaccines, science, government-affiliated health institutions and oneself. As respondents weighed whether to get vaccinated, they consistently conveyed, oftentimes via a personal narrative, how they found themselves contending with competing narratives that were shaped by emotional responses to the broader context.

**Fig. 2:**
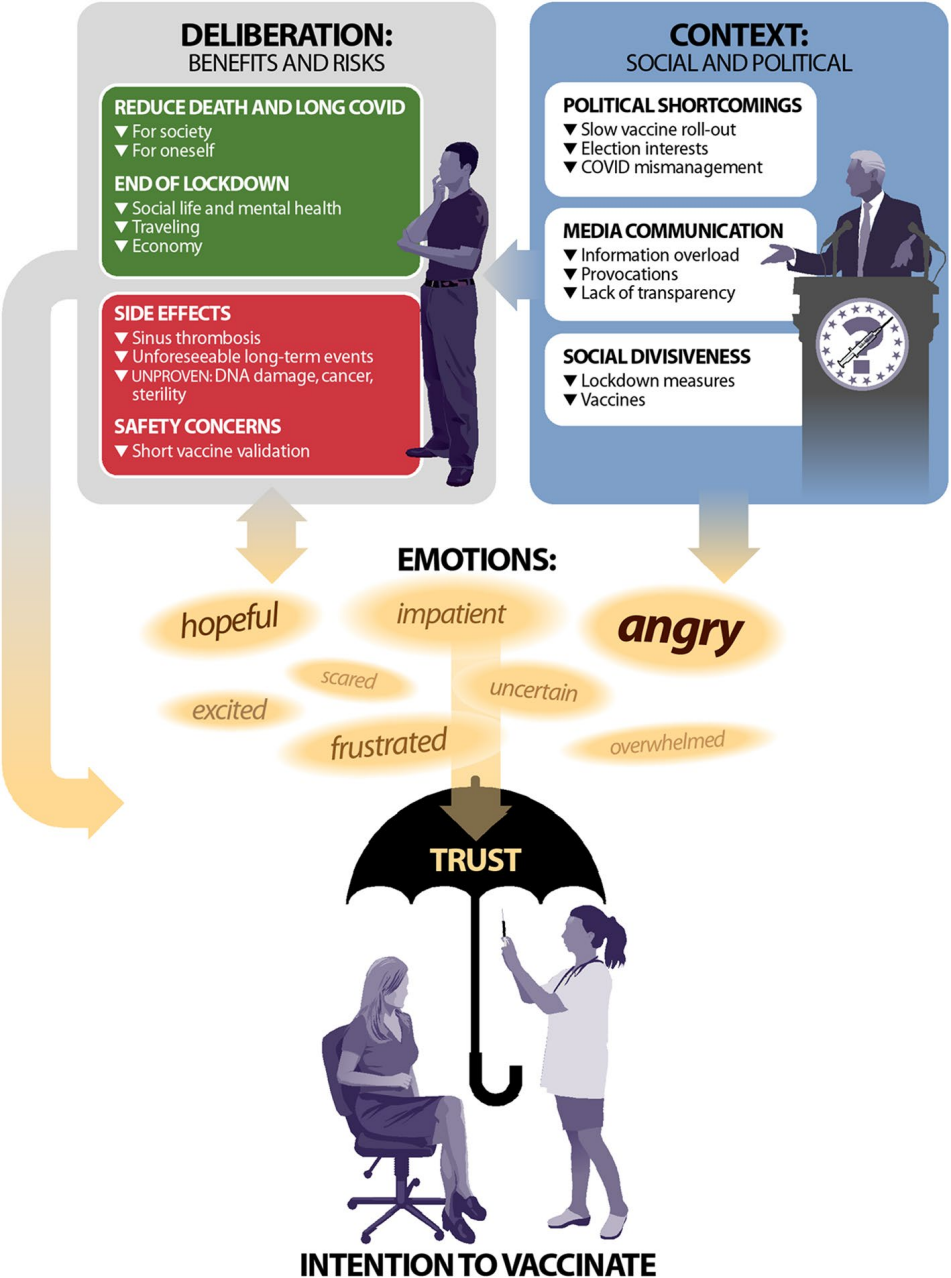
DCET framework of COVID-19 vaccination decision-making. Note 1: Long COVID is defined as “*signs and symptoms that continue or develop after acute COVID-19. The term includes ongoing symptomatic COVID-19 (4 to 12 weeks) and Post-COVID-19 syndrome (12 weeks or more)*” [75]. Note 2: Different font sizes of emotions represent the depth and nuance with which they were emphasized in interviews

Drawing upon these narratives, we developed the DCET framework (**D**eliberation, **C**ontext, **E**motion, **T**rust) of COVID-19 vaccination decision-making (see Fig. 2). We present our results following the flow of this figure. First, we outline deliberation (see section 3.1) wherein respondents, usually as a first step, weighed benefits and risks of vaccines. Second, we illustrate how these considerations were informed by contextual realities (see section 3.2) including political and interpersonal factors of COVID-19 and vaccines in Germany. Deliberation and context converged to spark a range of emotions (see section 3.3) about vaccines and vaccination, which in turn could lead to further, more personal deliberation (particularly regarding vaccine side effects). Although negative emotions were highly salient in participants’ narratives, they did not necessarily impact vaccination decisions in cases when respondents reported having trust (see section 3.4) in science, doctors, experts, and/or in their own capability to process information.

### Deliberation about benefits versus risks

When asked about opinions, experiences and attitudes on COVID-19 vaccines, respondents routinely illustrated how they weighed reasons for and against vaccination, highlighting internal debates about the risks of COVID-19 versus risks associated with vaccines, and navigating these fears to make decisions for or against vaccination uptake:

*“The tests before approval were less comprehensive than usual … but I think concerning the risks of getting blood clots compared to the risk of the pandemic … one is significantly smaller than the other. So I have little reservations, I’d be willing to take the risk.”* (female, 30 years).

#### Benefits of vaccines

Respondents generally described the benefits of vaccines as outweighing drawbacks. Vaccines were consistently highlighted as a valuable means to reduce suffering or mortality on a societal level, to end lockdowns and to resume normalcy. When describing the utility of vaccines, respondents often invoked a sense of doing what was in the best interest of society and recalled images of *“intensive care units with these completely helpless, profoundly sick, dying people”* (female, 61 years). Respondents, particularly younger respondents, perceived their own COVID-19-associated risks as minimal, but viewed vaccines as a means to protect vulnerable individuals within their family or social circle:

*“I was never really worried about myself, but more about harming others by unintentionally infecting those around me.”* (female, 30 years).

With similar frequency though less intensity, respondents described how vaccines would offer a means to end lockdowns, to meet friends and family, to engage in social and cultural activities, and to travel. As one young man said, *“the only relief for me would be to finally have a social life again.”* (male, 27 years). Some respondents described how social distancing and a constant fear of catching COVID-19 were detrimental to mental health, and others described the need to re-boot the German economy – in both cases highlighting vaccination as a means to address these issues. With less intensity and frequency, respondents described how a vaccine would mitigate concerns regarding problems associated with a moderate COVID-19 infection including loss of smell and long COVID (see legend of Fig. 2 for a definition). Respondents who had pre-existing conditions (diabetes, multiple sclerosis, lung diseases, etc.) perceived a high personal risk of severe illness from COVID-19 and viewed being vaccinated as *“a matter of life or death”* (male, 54 years).

#### Risks of vaccines

While respondents predominantly argued that the benefits of vaccination outweighed the risks, reservations centered on short-term side effects (namely, sinus thrombosis but also a high fever), broad safety concerns (due to a validation process described as unusually fast or *“immature”* [female, 61 years]) and potential long-term side effects (sterility, DNA-damage, or cancer). As one respondent said: *“I’ve heard that it causes blood clots and I don’t … even if they say [the risk is] really minimal that minimal might fall within me.”* (male, 37 years). Respondents described concerns such as being treated like a *“Versuchskaninchen”* [lit.: “*trial rabbit*”, sem.:*“guinea pig”*] (female, 57 years) by scientists *“experimenting with genetics”* (female, 21 years), with one older respondent recalling the thalidomide scandal in the 1960s as a cautionary tale^1^ [40].

#### Vaccine hesitancy regarding deliberation

Despite reservations, most respondents said they would ultimately get the vaccine, but some hesitated and described getting vaccinated as *“a basically frightening”* (female, 61 years) experience, best undertaken only after *“seeing how others respond to it”* (male, 27 years), and underscoring that they were concerned about the vaccine not preventing virus spread. Several respondents wondered aloud who would be accountable in the event of negative, vaccine-induced health consequences and whether vaccines might trigger or amplify mutations.

Two respondents conveyed outright rejection of COVID-19 vaccines. They described the risk of severe COVID-19 infection as minimal for younger people and lamented that information about the virus appeared *“fear-focused and detached from reality” (*male, 31 years). In their view, vaccines were generally dangerous (*“I don’t trust the procedures.”* female, 21 years) and there appeared to be no institutional recourse in the event of vaccine-related injury. Both respondents highlighted how they had grown up in a non-German context, and how they today partially relied on information they received from contexts outside of Germany, particularly regarding the potential harm of vaccines and how one’s own immune system was naturally capable of overcoming disease.

### Social and political context

Although not explicitly asked in interview guides, each respondent lamented some facet of the current social and political context in which COVID-19 vaccines were discussed, debated, promoted, and presented by politicians, journalists, or members of society.

#### Political shortcomings

Respondents described how, despite *“many well-performing public bodies”* (female, 51 years) in Germany, vaccine-related mismanagement (e.g. incomprehensible online registration portals, complex admission procedures within vaccination centers, or an inability to receive vaccines within family doctors’ offices) was *“rather problematic”* (female, 30 years) and served as one more example of political incompetence, thereby *“trying the limits of people’s patience”* (female, 51 years). Respondents decried the government’s vaccine purchase and supply chain process as *“abysmal”* (male, 57 years), *“wandering”* (male, 61 years), and *“amateurish”* (male, 57 years), leading to sentiments *“that [vaccination roll-out] is faster everywhere in the world, compared to us”* (male, 31 years). Respondents described how vaccines became a topic that seemed to serve political self-interest or a desire to expand personal clout in an election year:

*“How [vaccines] are being communicated to the public is fatal. And even beyond vaccines, there’s the lockdown measures, with politicians making promises they cannot keep. They have self-serving interests, probably because of the upcoming elections.”* (female, 30 years).

#### Media communication

Respondents also uttered dissatisfaction with how information was presented in news media. Divergent news on vaccines lead to a sense that journalists were intent on *“emotionally arousing”* (female, 37 years) reporting, *“ripping facts out of context”* (female, 58 years), *“showboating”* (*“Effekthascherei”,* female, 58 years), sowing stories that foment panic (*“Panikmache”,* female, 30 years), creating *“mass hysteria”* (female, 61 years), or encouraging broader social division related to COVID-19 vaccines. One woman explained that her negative opinion about one specific vaccine was *“triggered by the media, because you hear a lot more negative things about AstraZeneca than about the other vaccines. And you have to build your opinion based on what you hear, there is no other option.”* (female, 52 years). Respondents wished for more clarity and a greater sense of responsibility on behalf of the media concerning vaccination news, comparing the task to a mother who *“has a responsibility, and [I] must choose my words when I want to deliver a message.”* (female, 58 years).

#### Social divisiveness

Along with the political and media climate, respondents lamented the manner in which extended lockdowns and social distancing measures in Germany underpinned societal divisiveness and seemed to widen tensions between those in favor of and those opposed to COVID-19 vaccines. Respondents shared firsthand experiences of public conflict as those holding differing views on masks, vaccines, or social distancing engaged in arguments in public transportation, on the street, or online. Respondents who favored vaccines described a desire for expanded freedom for the vaccinated, and outlined frustration toward those who were unvaccinated, for whom they had *“very little understanding”* (female, 51 years). Unvaccinated people were further described as *“egotistical”* (female, 55 years) and “*careless*” (female, 51 years), and as inclined to *“demonize everything”* (male, 61 years), while society should instead focus on *“what it is actually about – that this vaccine is our hope”* (male, 31 years). Respondents who expressed outright objection to vaccination feared mandatory vaccines and indicated that they *“would be more open towards”* (male, 31 years) vaccination if it was voluntary.

### Emotions

Emotions emerged as a layer through which deliberation (benefits versus risks) and contextual factors (political, medial, and social) were filtered when making a decision about vaccines. Some participants voiced their desire to make a purely rational decision, without letting emotions influence their vaccination intention (see direct arrow from Deliberation to Trust in Fig. 2). In most of such cases, however, emotions were nevertheless verbally or non-verbally expressed. The dominant emotion associated with COVID-19 vaccines or vaccination in general across interviews, regardless of age or gender, was anger coupled with impatience, frustration, insecurity, or exasperation primarily directed at the slow roll-out of vaccines and other contextual factors, voicing concerns that *“it will take a hundred years until it’s my turn to get vaccinated”* (male, 20 years) and that *“that’s the biggest misstep I accuse our government of”* (male, 61 years). A young woman, who had been very hopeful about the vaccines, felt *“helplessness, fury, because you are forced to spend many more months of your life indoors without leisure time activities”* (female, 30 years). Respondents also fumed about how vaccines were presented in the media (particularly with regards to a perceived exaggeration of side effects such as sinus thrombosis caused by the AstraZeneca vaccine), and how vaccines were becoming another topic that was tearing society apart. Some respondents anticipated that provocative headlines and divisive news might lead to feeling *“insecure”* (female, 29 years) or *“confused”* (female, 21 years) about which vaccination information to believe, with one respondent conveying the feeling of being *“beaten to death by information”* (female, 51 years). Some respondents described feeling primed by the media to take a negative view (*“it sows in your mind some doubts towards it”,* male, 37 years), with one respondent stating:

*“If there is so much squabbling [German: “Hick Hack”] about a vaccine, you just can’t have a positive feeling. And there might be many who tell me: ‘Well, a few have died but it [the vaccine] has many positive aspects.’ Nevertheless, it remains an uneasy feeling.”* (female, 57 years).

#### Vaccine hesitancy regarding emotions

As highlighted earlier, the vast majority of respondents described their intention to get vaccinated (once a vaccine was available), and expressed a range of positive emotions and phrases to describe vaccines, such as *“ray of hope”* [German: *“Lichtblick”*] (male, 31 years), feeling *“happy to get vaccinated”* (male, 37 years) or *“relieved”* (female, 58 years) and able to feel safe again: *“What would it mean to catch COVID-19? Would I get severely ill? Would I die? Well, I’ll put it like this: I hope that this fear will decrease substantially when many people get vaccinated.”* (female, 51 years). Those respondents for whom risks of vaccines outweighed benefits described feeling “*ambivalent*” (female, 51 years), “*an inner apprehension*” (female, 21 years), or “*skepticism*” (female, 52 years) toward vaccination, and they wondered aloud: *“What are we exposing ourselves to, as world-population? […] Well, that’s my gut feeling.”* (female, 51 years).

### Trust

Almost all respondents outlined frustration with the COVID-19 response, concerns about the rapid pace of vaccine development, or exasperation with the government’s approach to vaccine distribution. Yet, when asked how these factors influenced decisions to vaccinate, respondents answered with phrases such as *“not at all”* (female, 55 years) or *“it is independent”* (female, 57 years). Upon further probing, respondents described how trust – in science and the scientific process, in doctors, institutions, media, or oneself (see Table 2 for key quotes with regards to the different trusted entities as emerging from respondents’ accounts) – safeguarded their vaccination intention or tipped the balance in favor of vaccination even in light of disheartening or unsettling contextual factors:

*“I had some reservations against the mRNA vaccine, as it hasn’t been used before. But well, I think the trust in the EMA [European Medicines Agency] regarding this ... yes, is stronger. What other than trust could it be? I think nobody knows what is going to happen in 20 years, whether we will light up green or something. Nobody can know that, I’m aware of this.”* (female, 58 years).

**Table 2:** Representative quotes on trusted entities related to vaccines and science

Trusted Entities	Quote
**Inherent value of vaccines and the scientific processes to develop them** • Vaccination as a useful and safe means to protect against infection. • Vaccine developers as conscientious and capable of inventing vaccines that work, ensuring safety and efficacy. • Short time of vaccination development and validation due to available funding, without compromising safety.	*“I also have lived in countries where having a vaccine is really important […] newborn babies for instance have longer lives because of that, which I have seen, many of them would have died before that. So, on that perspective I have never had [a desire to] mess with vaccines and [I have] seen the importance of them.”* (male, 37 years). *“Well, I believe in technology and science. And even if I don’t understand every detail, I have a basic level of trust and I think: Yes, awesome, they have a new idea, there’s a new technology, this is amazing. And well, yes, that will work.”* (male, 56 years).
**Regulatory processes and bodies that assess vaccine safety** • Appreciation of audit processes in the EU and Germany (e.g., EMA, RKI, PEI) as careful and transparent. • Acknowledgment of the likelihood of side effects but expecting that they will be closely monitored.	*“As soon as it’s my turn, I am going to get the shot (laughs), no matter which kind of vaccine because I assume that they have all been audited in advance – especially here in Germany, I guess – so that they are all safe and effective, of course.”* (female, 51 years).
**Individual stakeholders involved in vaccination development, advocacy, and rollout** • Physicians as people to turn to with questions and insecurities regarding vaccines. • Public health experts (e.g., scientists on social media) as a credible and trustworthy source to explain vaccine related facts.	*“My family physician knows me, she knows ... well, how I tick. She knows my worries concerning COVID and, ahem, vaccines. Because I talked to her about this. And I often get the feeling you hear others saying: ‘I went to the vaccination center and they blew me off by saying that it’s going to be okay.’”* (female, 29 years).
**One’s own capability to find reliable information** • Own capability to understand scientific facts and to find credible information after facing divisive news on Covid-19 vaccines.	*“I observed many people saying: ‘I don’t want to hear anything more about it (vaccines), I don’t listen to the news anymore.’ So they detach themselves mentally completely, maybe because they can no longer bear it or they feel helpless? I can only speculate as to why that is the case. I can’t do that, I have to inform myself. So, I have to ... I’m that kind of person, I like to swim in front of the wave. I want to find my own way and not be a sheep that runs with the herd. Not my thing.”* (female, 58 years).

The most trusted authorities indicated by respondents were scientific and medical institutions responsible for reviewing and validating vaccines in Germany. Even respondents who were skeptical about foreign vaccines (such as Russia’s Sputnik V) expressed that they would have confidence in them if German supervisory authorities decided to approve them. These trusted institutions included government-affiliated scientific institutions such as the European Medical Agency (EMA), the Robert Koch Institute (RKI, Germany’s main public health agency), and the Paul Ehrlich Institute (PEI, Germany’s Federal Institute for Vaccines and Biomedicines), while political actors such as elected government officials were much less trusted:

*“All that happens on a scientific level, like the EMA on the EU-level, the PEI in Germany – I trust completely. On a scale from 1-10, that’s a 10. But this goes into a minus range for federal- or state-level politics. So, I see vaccines very positively, but the way we handle them is evidence of incapacity* [German: “*Armutszeugnis*”].” (male, 37 years).

In general, respondents described feeling *“very inclined toward science”* (female, 55 years), scientists, and family physicians. The latter were particularly emphasized by several respondents because they expected family physicians to be beholden to their patients, to have a longstanding relationship with their patients, and to better understand patients’ medical histories and unique concerns or priorities in relation to medical information. Respondents also mentioned scientists as role models, naming prominent figures such as Dr. Mai Thi Nguyen Kim (a German chemist who focuses on science education via YouTube) or Prof. Dr. Christian Drosten (a German virologist and expert consultant of the government, who hosted an educational podcast over large parts of the pandemic), as well as scientists or experts with whom they personally interacted in their everyday environment. One student, for example, recounted how her professor helped her feel less insecure about vaccines by explaining how to interpret numbers about risks.

Finally, several respondents expressed trust in their own ability to sift through divisive or misleading media reports, and to better identify trustworthy information by reading scientific evidence themselves, particularly as a means to gauge risk:

*“I am a science student and, well, I know, to some extent at least, how to make sense of things that were reported in a misleading way. But I guess that most people are not familiar with this field and do not know where to find which information or which information to believe.”* (female, 37 years).

#### Vaccine hesitancy or refusal regarding deficits in trust

While the above examples highlight how high trust has protective qualities for vaccine uptake when facing negative emotions, some respondents also described how trust deficits facilitate the opposite consequence. Some respondents raised doubts regarding the general honesty of governmental stakeholders and media or explained how they could not find vaccine related information they were searching for online, suggesting that relevant facts about vaccines have been *“hidden”* or “*covered up”* (female, 21 years). The resulting insecurities prepared the ground to vaccine hesitancy: *“This creates extreme mistrust [...] and then it’s not a long way towards becoming vaccine hesitant, anti-vaccinator, conspiracy theorist.”* (male, 54 years).

## Discussion

To the best of our knowledge, this study is among the first to present in-depth qualitative research on COVID-19 vaccine decision-making in Germany. Respondents conveyed personal deliberation for and against vaccination as a proxy for weighing the risk of COVID-19 against the risks of vaccines, the contextual factors in which vaccines are currently being rolled out, and the emotions sparked by these considerations. We found trust in vaccines, science, and the health system to be key protective factors facilitating the intention to get vaccinated even in adverse situations. However, a loss in trust could potentially threaten the willingness to take up vaccines.

Our findings contribute to the ongoing debate on COVID-19 vaccination willingness and guidelines for vaccination rollout. Respondents in our study perceived vaccine rollout in Germany as suboptimal. These results reflect reports on growing dissatisfaction among the German public due to limited vaccination supply at the beginning of 2021 [32], anti-vaccinators becoming more vocal in the public discourse, and growing criticism of COVID-19 measures in general [30]. Whereas our sample in general showed high vaccination willingness, we found that media news regarding adverse events sparked substantial concerns, which mirrors earlier findings about influenza vaccination in Italy [41] and hepatitis B vaccines in China [42] – cases in which contradictory information and news about adverse events resulted in decreased vaccine uptake. Similarly, themes emerging from our study resonate with previous vaccine literature from Germany, including the relevance of hopes to protect oneself and others [43] or of (unfulfilled) desires for neutral information [44]. The relevance of safety concerns and (mis) information emerging from our work mirrors previous findings on an international level both with regards to COVID-19 vaccines [13, 22] and other diseases such as human papillomavirus [45–48], measles-mumps-rubella [49], influenza [50, 51], and polio [52].

Vaccine hesitancy poses major challenges to health systems globally, and several models have been developed to investigate and explain the phenomenon [8, 9]. A number of reasons for COVID-19 vaccine hesitancy in Germany identified in our study have been described in the context of existing theories such as the 5C model [8]: The aspect of “deliberation” in our work mirrors facets of calculation (weighing benefits versus risks), collective responsibility (protection of others), and complacency (risk of disease) within the 5C model. Our findings regarding “context” overlap with constraints (external factors that inhibit getting vaccinated) in the 5C model. “Trust” overlaps with aspects of confidence (trust in providers and health-care systems), and “trust in oneself” might include aspects of calculation (e.g. intensive information seeking). However, a majority of previous models were built based on vaccinations against diseases that existed for a longer time. In contrast, the COVID-19 pandemic posed novel challenges with strenuous contextual and social implications (such as lockdowns and high mortality). We therefore opted for developing a model that acknowledges these unique contextual factors and their impact on the individual beyond the way they are conceptualized in established models.

The framework inductively emerging from our data overlaps in several facets with other theories, most pointedly the Risk as Feelings Theory, which argues that risk perception in decision-making is influenced by an interplay of cognitive evaluation and affect, including anticipatory emotions of perceived risks and background mood [53]. One domain of the Risk as Feelings Theory that also featured prominently in our study and informs ongoing discourse related to vaccination involves emotion. Research has found that emotions and unconscious perceptions of negative messages influence vaccination decisions [54], and fear was identified as a more important driver for vaccine uptake than objective deliberations on risk (with regard to both vaccine and disease) [55, 56]. Additionally, decreases in vaccine confidence [7] have been shown to correlate with mistrust of the government in various settings [9, 57, 58].

Based on this evidence and recent data highlighting that trust in the German government and traditional media has decreased since February 2021 [29], a decrease in vaccination willingness would be likely. However, a majority of respondents in our study were inclined to get vaccinated despite negative emotional perceptions of contextual factors. As one reason, strong arguments in favor of vaccination seem to have outweighed fears and frustrations. Additionally, in many cases, negative emotions were attenuated by a high level of trust not only in vaccination as a concept, but also in institutions or sources of information, which adds to ongoing debates in the literature about the relation between trust and affect within risk-related decision-making [59]. Further, this finding underscores the seminal role of trust in science and researchers as a driver for vaccine uptake as highlighted in the international literature [60, 61]. Our findings align with conceptualizations describing trust as a concept people rely on in times of information absence or insecurity [62], resting upon an emotional basis [63]. This supports considerations of trust as superordinate meta-emotion [64] and, in line with our findings, we suggest considering it as an additional element within established frameworks such as the Risk as Feelings Theory when considering highly emotionalized and debated topics such as vaccination decision-making.

Our findings have implications for health policymaking. In reaction to mounting insecurities, respondents in our study called for transparent and honest information and communication, which can be considered a pathway to (re-)gaining trust. To this end, authors have recommended avoiding information overload [65] and circumventing integration of vaccines into political debate [66]. In the light of our findings, this sparks conversations about how to ensure transparency without overwhelming positively inclined individuals or raising insecurities regarding who to trust. We see potential in more pointed engagement with scientists, who were described as highly trusted among our respondents especially when compared to elected officials. Campaigns involving public participation and focusing on timely answering of public concerns, have previously been set in motion for COVID-19 [67] and could be successful tools to address upcoming fears, to gain trust [68], and to guide people in their vaccination decision-making.

Furthermore, we argue for strengthening the role of family physicians in vaccine education, which has been proven to be effective for other vaccines [69] and recently for COVID-19 related beliefs and practices [70]. Family physicians often have longstanding, trusting relationships with their patients [71], and they serve as role-models [72]. Such established relationships hold potential to address fears and emotional insecurities regarding vaccines, and to clarify divisive messages in a timely and personal setting. Our data further revealed that intentions to protect weaker individuals of society drove vaccination intention, especially in younger respondents. Thus, including messages about altruism and perceptions about vaccination as a social responsibility may bolster vaccination rates [73].

We collected our data amid a key phase of Germany’s COVID-19 vaccination rollout, when increasing COVID-19 incidence and the temporary suspension of one approved vaccine sparked many insecurities. This makes our work a unique and timely contribution not only to the current COVID-19 vaccination rollout, but also to literature on the effects of acute vaccine scares on vaccination intention. However, this study also has limitations. As COVID-19 vaccination is heavily encouraged in Germany, a social desirability bias may have resulted in respondents withholding opinions, criticism, or emotions. Furthermore, respondents were above average educated and no respondent over the age of 61 years answered our invitation for participation via Prolific Ltd. To improve the representation of older persons in online research, we recommend that future research combines, for example, analog invitation letters with subsequent online interviews. We urge further research before generalizing our findings. In our study, stark differences between subgroups such as gender, age, or ethnicity were limited. This speaks for the broad relevance and shared experiences across socio-demographic groups but - due to the small sample size inherent in qualitative research - does not rule out that such differences might influence the vaccination decision-making process. To systematically investigate how socio-demographic factors might influence vaccination decisions, the DCET framework might serve as the theoretical foundation for future research.

Our data revealed four main factors influencing COVID-19 vaccination decision-making in an emotionally charged context. Respondents who stated that they would take up a future vaccine when it became available could be categorized as vaccine accepting based on international standards [7]. Vaccine hesitancy, according to the definition [7] refers to a situation where vaccines are rejected despite their availability. As COVID-19 vaccination was not yet widely available at the time of data-collection, respondents were still in a decision-making phase without having agency to enact their decision. The DCET framework applies to the decision-making process across the vaccine hesitancy spectrum including accepting, hesitant and refusing perspectives. How the decision-making process of vaccine accepting, hesitant and refusing individuals may vary according to vaccine availability, and the extent to which the theoretical decision reflects subsequent vaccination rates may differ. We encourage future research to systematically investigate dynamics of vaccine decision making, including studies that quantitatively examine the relative importance and interrelatedness of DCET categories. Such research would allow refinement of evidence-based recommendations that could strengthen trust and bolster the effectiveness of future vaccination campaigns.

## Conclusion

High vaccination rates are needed to address the COVID-19 pandemic. In a highly challenging and emotionally charged context, high levels of trust can counterbalance negative affect or contra-arguments. We emphasize that the public needs to be informed transparently about scientific results in order to trust the process. At the same time, the public reacts sensitively to potentially unsettling messages about vaccine side effects. This research underpins the importance of thoughtful, but nonetheless honest and transparent communication of all actors in the health system as a step to preserve, extend, or re-gain trust in vaccines.

## Supplementary Information

**Additional file 1:.** Semi-structured, in depth-interview guide (German and English version).

## Data Availability

Due to the sensitive nature of qualitative data, datasets generated and/or analyzed during the study are not publicly available to ensure interviewees’ privacy. Data can be made available upon reasonable request on a case-by-case basis; please contact the corresponding author.

## Footnotes

1 Thalidomide was a sedative drug recommended to pregnant women from 1957 to 1962 due to assumed safety and its effectiveness against morning sickness. Uptake was particularly high in Western Germany where the drug was available over the counter. A few years later, cases of malformations in children whose mothers had taken the drug during pregnancy became evident [40].
