# Supplementary material for: Deliberation, context, emotion and trust – understanding the dynamics of adults’ COVID-19 vaccination decisions in Germany
Source: BMC Public Health. 2023 Jan 19;23:136. doi: 10.1186/s12889-022-14587-7 (PMC9850339; doi:10.1186/s12889-022-14587-7)
Supplement: Supplementary file 1 — Additional file 1:. Semi-structured, in depth-interview guide (German and English version). [file 12889_2022_14587_MOESM1_ESM.pdf]

Note: Qualitative interviewing is process in which certain topics mentioned by respondents will be further investigated on. The questions in this semi-structured guide represent a first guideline for all interviews. In each conversation, more detailed questions based on the interviewee's experiences may arise in order to understand the content more precisely. Over the course of the data collection, the questions evolved or more probes were added.

German original: Question Guide for In-Depth Interview

Preamble: Mein Name ist (*Interviewer Name*). Unser Interview heute ist Teil einer größeren Studie des Heidelberger Instituts für Global Health der Universität Heidelberg. In dieser Studie geht es um Präferenzen und Meinungen bezüglich potentieller Impfungen gegen COVID-19. Wir führen diese Studie in mehreren Ländern durch, um die Meinungen und Präferenzen der Einwohner:innen zu erfassen und Ideen und Sorgen zu diskutieren.

Alle Informationen, die Sie heute mit mir teilen, werden nur in pseudonymisierter Form verwendet, ein Rückschluss auf Sie als Person wird nicht möglich sein. Die Informationen, die Sie mit mir teilen, werden nur für die Erforschung zu COVID-19 Impfungen und wie diese die Erwartungen der Bürgerinnen und Bürger erfüllen können, verwendet. Dieses Interview wird etwa 45 Minuten dauern. Die Ethikkommission der Medizinischen Fakultät der Universität Heidelberg hat diese Studie begutachtet.

Statement zur informierten Einwilligung: (*Wird ein weiteres Mal vorgelesen und Einwilligung wird erfasst. Im Anschluss beginnt die Aufzeichnung*)

Teil A – COVID-19 Impfung

Preamble: Die Entwicklung und Verteilung einer COVID-19 Impfung ist eine der besten Chancen, die andauernde Pandemie zu beenden und Menschen auf der ganzen Welt gegen diese Krankheit zu schützen. Die Entwicklung einer Impfung dauert, und es gibt strenge Richtlinien für die Entwicklung: Die Impfung durchläuft vier Phasen. Zuerst wird die Impfung an Zellen, dann an Tieren getestet. In der dritten Phase wird die Impfung einigen wenigen Menschen verabreicht, um ihre Effekte zu beobachten, und dann vielen tausend Menschen um zu erfassen, wie gut

sie gegen die jeweilige Krankheit schützt. Nur wenn eine Impfung all Phasen erfolgreich durchlaufen hat, wird sie auch zugelassen. Wie Sie vielleicht gehört haben, haben einige Impfungen gegen COVID-19 bereits die Zulassung erhalten, viele weitere befinden sich noch in der Entwicklung.

1. Was denken Sie über die COVID-19 Impfung?
    a. Was haben Sie bisher über COVID-19 Impfung(en) gehört?
    b. Was denken Sie darüber?
    c. Was macht Ihnen Hoffnung in Bezug auf eine COVID-19 Impfung?
    d. Was macht Sie nervös in Bezug auf eine COVID-19 Impfung?

Für die nächsten Fragen, stellen Sie sich bitte vor Sie wären für die Entwicklung und Auslieferung einer COVID-19 Impfung verantwortlich. Stellen Sie sich vor, sie könnten zentrale Dinge bezüglich dieser Impfung entscheiden – wie sie aussehen sollte, wer sie bekommen sollte, und wie.

2. Wenn Sie eine COVID-19 Impfung entwickeln könnten, was wären im Idealfall ein paar Dinge, die Sie auf jeden Fall miteinbeziehen würden? Bitte beschreiben Sie sie mir so detailliert wie möglich (*längere Pause einbauen, den/die Teilnehmer/in eine Weile nachdenken lassen. Erst dann follow-up Fragen stellen*)
    a. Probes:
        i. Das ‚Aussehen' der Impfung (Farbe, Größe…)
        ii. Die Schutzwirkung/Effizienz der Impfung
        iii. Die Dauer des Schutzes der Impfung
        iv. Akzeptable vs. Inakzeptable Nebeneffekte
        v. Wie sollten Menschen von dieser Impfung erfahren?
        vi. Wer sollte die Impfung verabreichen, und wie sollte das passieren (Schluckimpfung, Spritze…)
        vii. Wo sollte die Impfung verabreicht werden?
        viii. Wer sollte priorisiert geimpft werden?

Ok, lassen Sie mich kurz zusammenfassen. Bitte unterbrechen Sie mich, falls ich etwas falsch verstanden habe oder ich einen Fehler mache. Ihre ideale Impfung würde XXX aussehen. Sie würde XXX stark gegen COVID-19 schützen. Nebeneffekte könnten XXX sein, aber nicht YYY. Die Menschen würden an Ihrer

Impfung mögen, dass sie XXX und YYY von ihr gehört haben. Die Menschen erhalten die Impfung XXX, und zuallererst würden YYY sie bekommen.

Teil B – Impfskepsis

Stellen Sie sich vor, dass manche Menschen die Impfung, die Sie entwickelt haben, nicht haben möchten.

3. Was würden Sie tun, um diese Menschen von Ihrer Impfung zu überzeugen?

Teil C – Abschluss

4. Gibt es sonst noch irgendwas, das Sie mit mir besprechen möchten?
5. Haben Sie noch offene Fragen oder Kommentare?

Herzlichen Dank für Ihre Zeit!

(translated) English version: Question Guide for In-Depth Interview

Preamble: I am *(interviewer's name)*. Our meeting today is part of a larger study which the Heidelberg Institute of Global Health, Heidelberg University, Germany conducts to better understand public preferences, beliefs and values regarding a potential COVID-19 vaccination. We conduct this study in several countries as a means to learn peoples' attitudes and preferences, and to address their ideas and concerns.

We are independent researchers. The information you share with us will only be used anonymously. The information you share with us will only be used to design vaccine-related research and interventions that meet the needs and preferences of citizens. Our interview will last around 45 minutes. We have obtained ethical clearance for this study from the ethical review board of the Medical Faculty at Heidelberg University, Germany.

Statement of informed consent: *(read out written informed consent form, obtain consent; Start tape recording if consent is granted).*

Part A – COVID-19 vaccination

Preamble: Developing a vaccine against COVID-19 is one of the best chances to end the ongoing pandemic, and protect people around the world against this disease. Developing a vaccine takes time, and there are strict guidelines in place regarding how a vaccine is developed: the vaccine testing process involve four phases which include to first test the vaccine on cells and animals, then on a few people to monitor its effects, and then on thousands of people to assess how well it protects against the disease. Only if a vaccine passes all phases successfully, it will be approved. As you might have heard, first vaccines already have been approved and dozens more are currently being developed.

1. Please share your thoughts about a COVID-19 vaccine.
    a. What, if anything, have you heard about a COVID-19 vaccine?
    b. How do you feel about it?
    c. What makes you feel hopeful about a COVID-19 vaccine?
    d. What makes you feel nervous about a COVID-19 vaccine?

For the next few questions, I want you to imagine that you are in charge of developing and introducing a COVID-19 vaccine. Imagine that you can decide important things about the vaccine. How it should look, who should get it and how that should happen.

2. If you could design the COVID-19 vaccine, what are some things you would make sure it has? Please describe it for me in as much detail as you can, and talk with me like you would with a friend. ***(allow long pause, let the respondent think about this for a while. only ask follow up questions if they seem confused)***
    a. Probes:
        i. The "look" of the vaccine (color, size, packaging)
        ii. The vaccine's efficacy to protect against COVID-19
        iii. The vaccine's duration to protect against COVID-19
        iv. Acceptable versus unacceptable side effects
        v. How do you think people should learn about the vaccine?
        vi. Who should give the vaccine, and how should the vaccine be administered (injection or mouth drops)?
        vii. Where do you like the vaccine to be delivered?
        viii. Who should get priority in getting the vaccine? How do you pick who gets it first?

OK, let me re-cap what you've said. Please interrupt me if I make a mistake or something about what I say isn't quite right. Your ideal vaccine will look like *XXX*. It will be *XXX* strong in protecting against COVID-19. Side effects can include *X* but not *Y*. People will feel good about your vaccine because they will have learned about it *HERE* and *THERE* and if they have questions, they can go *HERE*. When people need to get vaccinated, they will get it from *HERE*. The people who should get the vaccine first or early should be *XX* and the people who should not get it at all, if any, would be *XX*.

Part B – Vaccine hesitancy

Let's imagine that some of the people you approach with this vaccine do not want to get it. You have ***(Reiterate everything the respondent stated earlier about safety, who gives it, information, color, distribution)***.

3. What would you say or do to make these people feel better about your vaccine?

Part C – Conclusion

4. Is there anything we haven't talked about that you would like to discuss?
5. Do you have any additional comments?

Additional files:

Additional file 1.pdf: Semi-structured, in depth-interview guide (German and English version).
